# Hyperthermia differentially affects specific human stem cells and their differentiated derivatives

**DOI:** 10.1007/s13238-021-00887-y

**Published:** 2021-11-01

**Authors:** Si Wang, Fang Cheng, Qianzhao Ji, Moshi Song, Zeming Wu, Yiyuan Zhang, Zhejun Ji, Huyi Feng, Juan Carlos Izpisua Belmonte, Qi Zhou, Jing Qu, Wei Li, Guang-Hui Liu, Weiqi Zhang

**Affiliations:** 1 Advanced Innovation Center for Human Brain Protection, National Clinical Research Center for Geriatric Disorders, Xuanwu Hospital Capital Medical University, Beijing, 100053 China; 2 State Key Laboratory of Membrane Biology, Institute of Zoology, Chinese Academy of Sciences, Beijing, 100101 China; 3 State Key Laboratory of Stem Cell and Reproductive Biology, Institute of Zoology, Chinese Academy of Sciences, Beijing, 100101 China; 4 CAS Key Laboratory of Genomic and Precision Medicine, Beijing Institute of Genomics, Chinese Academy of Sciences, Beijing, 100101 China; 5 National Laboratory of Biomacromolecules, CAS Center for Excellence in Biomacromolecules, Institute of Biophysics, Chinese Academy of Sciences, Beijing, 100101 China; 6 Aging Translational Medicine Center, International Center for Aging and Cancer, Xuanwu Hospital, Capital Medical University, Beijing, 100053 China; 7 Institute for Stem Cell and Regeneration, Chinese Academy of Sciences, Beijing, 100101 China; 8 University of Chinese Academy of Sciences, Beijing, 100049 China; 9 China National Center for Bioinformation, Beijing, 100101 China; 10 Beijing Institute for Stem Cell and Regenerative Medicine, Beijing, 100101 China; 11 Gene Expression Laboratory, Salk Institute for Biological Studies, La Jolla, CA USA; 12 Chongqing Renji Hospital, University of Chinese Academy of Sciences, Chongqing, 400062 China

**Dear Editor,**

The human body operates optimally at a core temperature of 37 degrees Celsius. Homeostasis at this temperature is essential for cellular and physiological functions (Cheshire, 2016). However, infectious diseases, inflammation, injury, neoplasia, and elevated climate temperature can cause a regulated rise in body core temperature, i.e., fever (Pasikhova et al., 2017). Indeed, an acute or chronic increase in temperature leads to detrimental effects on vasculature by altering a number of indices of vascular structure and function (DuBose et al., 1998). In addition, fever during pregnancy has been associated with an increased risk of neurodevelopmental impairment and congenital heart disease in offspring (White et al., 2007; Xia et al., 2019). An in-depth understanding of the cellular and molecular responses to febrile temperature in major body organs and tissues is, therefore, of scientific and clinical importance. Here, we construct a systematic, transcriptional atlas of fever-range heat stress across pluripotent and adult stem cells as well as their derivatives. Using this approach, we identify the cell types that are most susceptible or resistant to heat stress, as well as the fever-induced changes in gene expression. Our data provide a useful resource as well as potential biomarkers and therapeutic targets for the diagnosis and treatment of fever-associated disorders.

Stem cells, including pluripotent stem cells and adult stem cells, are able to self-renew to maintain a stem cell pool, but also can be differentiated into specialized cells (Zakrzewski et al., 2019). Stem cell dysfunction, induced by internal or external stresses such as heat stress, may serve as a major contributor factor for the decline in tissue development, regeneration and homeostasis, ultimately contributing to a variety of diseases. Pluripotent stem cells include human embryonic stem cells (hESCs), which retain pluripotent properties *in vitro* and can differentiate into diverse cell types that include multipotent human mesenchymal stem cells (hMSCs) and human neural stem cells (hNSCs). In addition, hESCs can give rise to terminally differentiated cells such as human vascular endothelial cells (hVECs), human vascular smooth muscle cells (hVSMCs), human cardiomyocytes (hCMs), and human neurons (hNeurons) (Cheng et al., 2019; Yan et al., 2019; Li et al., 2020). Although heat stress has detrimental effects on a variety of organs, there is a lack of a systemic analysis of the cellular and molecular responses to heat stress in human stem cells and their derivatives, which may help reveal mechanisms relevant to fever-related stem cell dysfunction.

In this study, we systematically examined the effects of exposure to continuous febrile temperature on a variety of stem cells and terminally differentiated cells. First, we obtained and characterized the aforementioned cell types (Fig. 1A). hESCs expressed pluripotency markers OCT4, NANOG and SOX2 (Fig. S1A). hVECs, hVSMCs, hMSCs, hCMs and hNSCs were directly differentiated from hESCs, as confirmed by the expression of cell type-specific markers (Fig. S1B–F). Derived hVECs expressed VEC-specific markers CD31, CD144, and endothelial nitric oxide synthase (eNOS) (Fig. S1B). hVSMCs expressed SM22 and calponin as expected (Fig. S1C). hMSCs were positive for hMSC markers, including CD90, CD73, and CD105 and negative for non-hMSC markers including CD34, CD43, and CD45 (Fig. S1D). hCMs expressed the cardiomyocyte-specific marker cardiac troponin T (cTnT) (Fig. S1E). hNeurons were differentiated from SOX2, Nestin and PAX6-positive hNSCs (Fig. S1F) and expressed the neuron-specific marker MAP2 (Fig. S1G) (See SUPPLEMENTARY MATERIALS). Moreover, transcriptomic analysis revealed cell type-specific molecular signatures accordant with the function of each cell types. For instance, the genes specifically expressed in hVEC transcriptome were enriched for “vascular development”, hNSCs for “neurogenesis”, hNeurons for “synaptic signaling” (Fig. S1H), etc. Thus, we obtained and characterized a variety of human stem cells and their derivatives, providing a cellular platform for downstream analysis.

**Figure 1:**
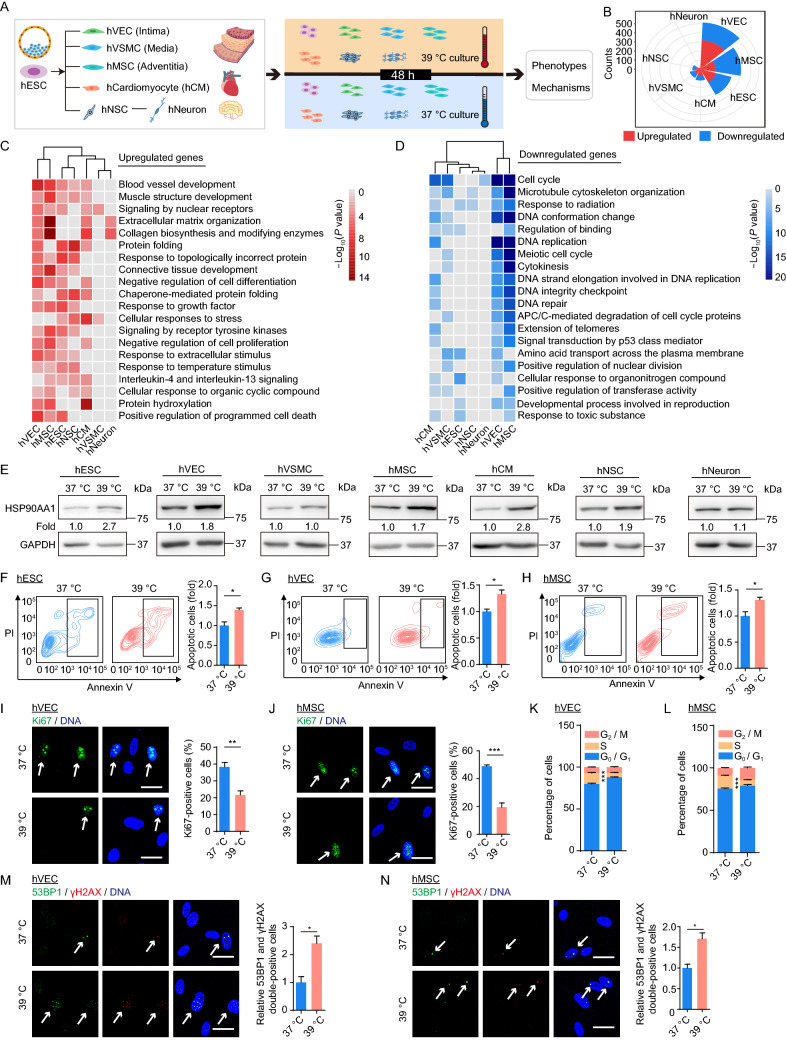
**Establishment of the transcriptional landscape of human stem cells and their derivatives under 39 °C heat stress.** (A) A schematic of the workflow. The febrile temperature experiments were carried out in hESCs at early passages (between 50–60 passages), in hVECs and hVSMCs at passage 2 or 3, in hMSCs at passage 4 or 5, in hNSCs at passage 6 or 7, in hCMs and hNeurons at approximate day 21 from differentiation. (B) Rose diagram showing the numbers of 39 °C hyperthermia-associated differentially expressed genes (hereafter referred as hyperthermia DEGs) in the indicated cell types. (C and D) GO term and pathway enrichment analysis based on upregulated (C) or downregulated (D) hyperthermia DEGs across seven cell types. The color key from red to gray (C), or from blue to gray (D) indicates *P* values from low to high, respectively. (E) Western blot analysis of HSP90AA1 expression across seven cell types under control (37 °C) and febrile temperature (39 °C) culture conditions. (F–H) Flow cytometric analysis of apoptosis in hESCs (F), hVECs (G) and hMSCs (H) under control (37 °C) and febrile temperature (39 °C) culture conditions. Data are shown as the mean ± SEM, *n* = 3; *P < 0.05. (I and J) Immunofluorescence analysis of Ki67 expression in hVECs (I) and hMSCs (J) under control (37 °C) and febrile temperature (39 °C) culture conditions. Data are shown as the mean ± SEM, *n* = 3, **P < 0.01, ***P < 0.001. Scale bar, 25 µm. (K and L) Flow cytometric analysis of cell cycle in hVECs (K) and hMSCs (L) under control (37 °C) and febrile temperature (39 °C) culture conditions. Data are shown as the mean ± SEM, *n* = 3, ***P < 0.001. (M and N) Immunofluorescence analysis of 53BP1 and γH2AX expression in hVECs (M) and hMSCs (N) under control (37 °C) and febrile temperature (39 °C) culture conditions. Relative 53BP1 and γH2AX double-positive cells were calculated and are shown as the mean ± SEM, *n* = 3, *P < 0.05. Scale bar, 25 µm

To elucidate the underlying molecular mechanisms linked to continuous febrile temperature (in a physiological sense) on various cell types, we performed genome-wide RNA-sequencing (RNA-seq) across seven cell types challenged by continuous maintenance at 39 °C for 48 h. High reproducibility of transcriptome expression profiles between replicates was confirmed by Euclidean distance analysis and principal component analysis (PCA) (Fig. S1I and S1J). hVECs, hMSCs, and hESCs were the most sensitive cell types to fever-range hyperthermia, based on the number of differentially expressed genes (DEGs) at 39 °C for 48 h (Figs. 1B and S1K; Table S1). 39 °C hyperthermia altered the expression of 514 genes in hVECs (317 upregulated and 197 downregulated), 402 genes in hMSCs (148 upregulated and 254 downregulated), and 319 genes in hESCs (161 upregulated and 158 downregulated) (Fig. S1K). hVECs and hMSCs represent major cell types found within the intima and adventitia of blood vessels, respectively (Yan et al., 2019). This implies that the vasculature is particularly susceptible to fever. hNeurons were least affected by 39 °C and had the smallest number of DEGs (10 upregulated and 7 downregulated) (Fig. S1K). These results suggest that, at least in the condition of 39 °C for 48 h , neurons may be more resistant to heat shock and resilient to maintain the stability of neural circuity.

Overall, we discovered that most DEGs linked to hyperthermia were lineage-specific or cell type-specific. Only a few DEGs were shared across all seven cell types (Fig. S2A). However, there were some shared Gene Ontology (GO) terms across most cell types (Fig. 1C and 1D; Table S1). For example, upregulated genes are linked to proteostasis (“protein folding”, “response to topologically incorrect protein”, “chaperone-mediated protein folding”) in all analyzed cell types except for hVSMCs and hNeurons (Fig. 1C; Table S1). Consistent with these results, we observed an increase in HSP90AA1 (a canonical heat shock protein (HSP)) in all cell types, other than hVSMCs and hNeurons, at 39 °C for 48 h (Fig. 1E), indicating the cells are indeed in a heat-stressed state (White et al., 2007; Lin et al., 2019). Moreover, we observed increased gene expression in the categories “response to temperature stimulus” and “positive regulation of programmed cell death” (*CLU*, *PHLDA3*) in hVECs, hMSCs, and hESCs (Figs. 1C and S2B), which was consistent with an increased frequency of apoptotic cells at 39 °C for 48 h (Fig. 1F–H). Notably, we found that hVECs and hMSCs displayed a similar molecular response to febrile temperature, evidenced by more commonly downregulated genes (53 genes) and functional terms (Figs. 1D and S2A). Specifically, the GO terms “cell cycle” and “DNA repair” were enriched among downregulated genes in hVECs and hMSCs upon 39 °C heat shock (Figs. 1D, S2C and S2D), consistent with a decline in Ki67-positive cells and S-phase cells (Fig. 1I–L), along with increased 53BP1 and γH2AX double-positive cells (Fig. 1M and 1N). Correspondingly, commonly downregulated genes associated with “cell cycle” were *MYBL2, KIF2C* and *CCNA2* (Fig. S2C), and genes related to “DNA repair” such as *MCM6*, *BRCA2* and *TRIP13* (Fig. S2D). In conclusion, our results demonstrate that fever reshapes the transcriptome landscape with major changes, including an elevated unfolded protein response, apoptosis, cell cycle arrest, and compromised DNA repair.

To better understand the specific effects of febrile temperature on diverse cell types, we analyzed hyperthermia-associated, cell type-specific DEGs (Fig. S3A). The largest numbers of upregulated/downregulated genes were observed in hVECs (251 upregulated) and hMSCs (162 downregulated) (Fig. S3A). Functional enrichment analysis of those genes highlighted a dysregulation of cell type-specific biological processes (Fig. 2A and 2B). For instance, upregulated genes in hESCs were enriched in “regulation of ossification” and “embryonic digit morphogenesis”. “angiogenesis” and “vascular development” were highly enriched among upregulated genes in hVECs. “extracellular matrix organization” and “skeletal system development” were enriched in hMSCs (Fig. 2A). The downregulated genes in hESCs were enriched in the terms “cell morphogenesis involved in differentiation” and “axon development”. “vascular process in circulatory system” was enriched among downregulated genes in hVECs, and “calcium ion transport” in hNSCs (Fig. 2B). Furthermore, to determine putative upstream factors that regulate heat shock-induced cell type-specific DEGs, we performed cis-regulatory motif analysis using RcisTarget. Although every cell type possessed cell type-specific genes, cis-regulatory motif analysis revealed a number of shared upstream regulators for both upregulated and downregulated genes (Fig. S3B and S3C). For example, cis-regulatory motif analysis revealed that the core transcription factors *GTF2F1* and *ARID3A*, as well as *E2F1* and *SRF*, were linked to upregulated or downregulated genes across most of the cell types, respectively (Fig. S3B and S3C). Collectively, our findings provide a comprehensive and valuable resource that deepens understanding of the molecular mechanisms underlying cell type-specific effects during febrile temperature exposure.

**Figure 2:**
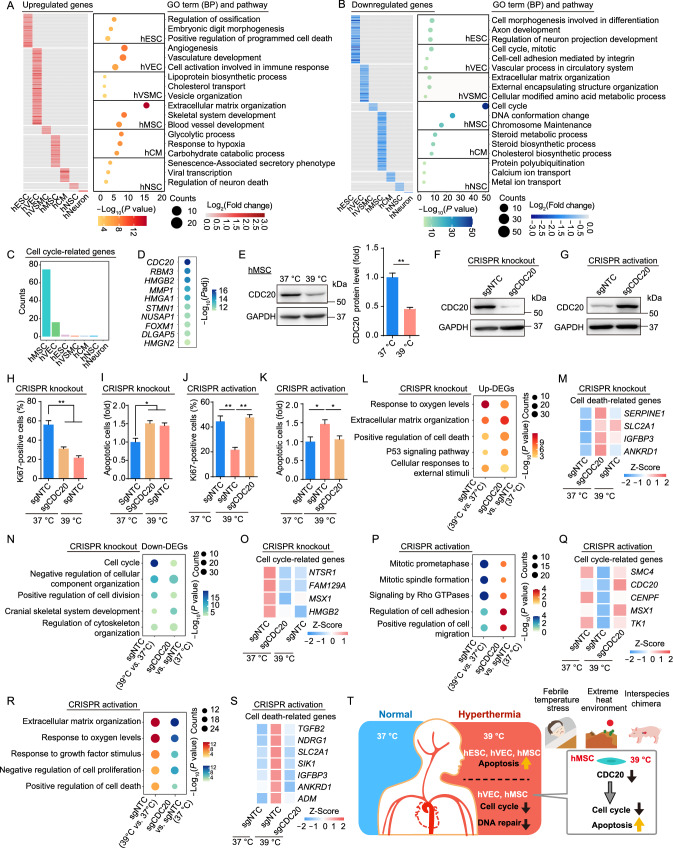
**Cell type-specific transcriptional signatures highlight the impaired cell proliferation of hMSCs upon heat stress shock.** (A and B) Heatmaps showing the relative transcriptional changes for cell type-specific upregulated (A) and downregulated (B) hyperthermia DEGs in seven cell types. Representative Gene Ontology (GO) terms for each set of DEGs are shown to the right. (C) Bar plot showing the counts of cell type-specific hyperthermia DEGs related to cell cycle. (D) Dot plot showing the top ten significantly downregulated genes of hyperthermia DEGs in hMSCs. (E) Left, western blot analysis showing the expression of CDC20 in hMSCs under control (37 °C) and febrile temperature (39 °C) culture conditions. GAPDH was used as loading control. Right, relative protein level of CDC20 was calculated and is shown as the mean ± SEM, *n* = 3, **P < 0.01. (F and G) Western blot analysis of CDC20 protein in hMSCs transduced with sgNTC or CDC20-targeting knockout sgRNA (F) or CDC20-targeting activation sgRNA (G). GAPDH was used as the loading control. (H) Immunofluorescence analysis of Ki67 in hMSCs transduced with sgNTC or CDC20-targeting knockout sgRNA under control (37 °C) and febrile temperature (39 °C) culture conditions. Data are presented as the mean ± SEMs, *n* = 3. **P < 0.01. (I) Flow cytometric analysis of apoptosis in hMSCs transduced with sgNTC or CDC20-targeting knockout sgRNA under control (37 °C) and febrile temperature (39 °C) culture conditions. Data are presented as the mean ± SEMs, *n* = 3. *P < 0.05. (J) Immunofluorescence analysis of Ki67 in hMSCs transduced with sgNTC or CDC20-targeting activation sgRNA under control (37 °C) and febrile temperature (39 °C) culture conditions. Data are presented as the mean ± SEMs, *n* = 3. **P < 0.01. (K) Flow cytometric analysis of apoptosis in hMSCs transduced with sgNTC or CDC20-targeting activation sgRNA under control (37 °C) and febrile temperature (39 °C) culture conditions. Data are presented as the mean ± SEMs, *n* = 6. *P < 0.05. (L) Representative shared GO terms enriched for the upregulated hyperthermia DEGs and upregulated DEGs upon knockdown of CDC20 in hMSCs. (M) Heatmap showing the similar expression changes of cell death-related genes upon heat stress shock and knockdown of CDC20 in hMSCs. (N) Representative shared GO terms enriched for the downregulated hyperthermia DEGs and downregulated DEGs upon CDC20 knockdown. (O) Heatmap showing the similar expression changes of cell cycle-related genes upon heat stress shock and knockdown of CDC20 in hMSCs. (P) Representative rescued GO terms enriched for the downregulated hyperthermia DEGs and upregulated DEGs upon activation of CDC20 in hMSCs. (Q) Heatmap showing the expression of cell cycle-related genes that were downregulated by heat stress shock but rescued by activation of CDC20 in hMSCs. (R) Representative rescued GO terms enriched for the upregulated hyperthermia DEGs and downregulated DEGs upon activation of CDC20 in hMSCs. (S) Heatmap showing the expression of cell death-related genes that were upregulated by heat stress shock but rescued upon the activation of CDC20 in hMSCs. (T) A schematic illustration showing the phenotypic and transcriptomic characteristics of diverse human stem cells and their derivatives upon 39 °C heat stress shock

hMSCs, a kind of adult stem cells that are widely distributed in the body, play an essential role in cell replenishment and regeneration of damaged tissues via continuous self-renewal and differentiation. In line with their defects in mitosis upon febril temperature (Fig. 1J and 1L), we observed that the expression levels of cell cycle-related genes are markedly reduced in hMSCs (Fig. S3D), and the number (75) and the percentage (46.3%) of downregulated genes associated with cell cycle regulation were the highest in hMSCs (Figs. 2C and S3E). These data suggest that self-renewal is substantially compromised in hMSCs upon heat stress.

To explore key factors contributing to severe cell cycle arrest in hMSCs upon febrile temperature, we analyzed the top 10 downregulated genes, and found 40% of them were cell cycle-related genes (Fig. 2D). *CDC20* is the top downregulated gene in hMSCs after exposure to 39 °C (Fig. 2D), as further verified by Western blot (Fig. 2E). Next, we investigated whether CDC20 knockdown or activation can mimic or rescue the phenotypes induced by hyperthermia in hMSCs (Fig. S3F). We depressed CDC20 expression using the CRISPR-Cas9-mediated gene knockout system and induced endogenous CDC20 expression in hMSCs using the CRISPR-dCas9 transcriptional activation system. The activation system reflects a physiological state more closely than expression from an exogenous promoter (Joung et al., 2017). Knockdown and activation efficiencies were validated by Western blot (Fig. 2F and 2G). Knockdown of CDC20 decreased cell proliferation, consistent with the reduced cell propagation seen at 39 °C (Figs. 2H and S3G). Moreover, we observed elevated levels of apoptosis after CDC20 silencing in hMSCs, also consistent with the heat shock phenotype (Figs. 2I and S3H). CDC20 activation increased proliferation and alleviated the 39 °C hyperthermia-induced cellular apoptosis in hMSCs (Figs. 2J, 2K, S3I and S3J). RNA-seq analysis also demonstrated that CDC20 knockdown resulted in a gene expression profile resembling the 39 °C heat shock in hMSCs (Fig. S3K–L; Table S2). This includes upregulated genes related to “positive regulation of cell death” (e.g., *IGFBP3* and *ANKRD1*) and “cellular responses to external stimulus” (Fig. 2L and 2M; Table S2), as well as to downregulated genes related to “cell cycle” and “positive regulation of cell division” (e.g., *HMGB2*) (Fig. 2N and 2O; Table S2). By contrast, CDC20 activation reversed the transcriptional profile under heat shock stress (Fig. S3M and S3N; Table S2). For example, the expression of hyperthermia-associated downregulated genes related to “mitotic prometaphase” and “mitotic spindle formation” (e.g., *SMC4* and *CENPF*) recovered after CDC20 activation in hMSCs (Fig. 2P and 2Q; Table S2). In contrast, heat stress-induced genes involved in “positive regulation of cell death” (e.g., *IGFBP3* and *ANKRD1*) were attenuated in CDC20-activated hMSCs (Fig. 2R and 2S; Table S2). In conclusion, our findings demonstrate that downregulation of *CDC20* is potentially a major driver that mediates the detrimental effects of fever in hMSCs, including compromised proliferation and increased apoptosis.

Fever is a common phenomenon in various infectious and immune-related diseases, but its influence and underlying molecular regulatory signature in stem cells and cells from different lineages remain poorly understood. Here, we, for the first time, systematically examined the transcriptional landscape reestablished by fever across pluripotent and adult stem cells along with their various derivatives. We provide novel insights into the common and cell type-specific deleterious effects upon heat stress. Our comprehensive transcriptomic analysis demonstrated that pluripotent hESCs, adult stem cell hMSCs, and terminally differentiated hVECs were differentially affected by physiological heat shock. Major features of the febrile response included increases in the unfolded protein response and apoptosis, as well as cell cycle arrest and impaired DNA repair. Furthermore, CDC20 was identified as a core cell cycle regulator whose downregulation was responsible for impaired cell proliferation in heat-stressed hMSCs. In summary, our data provide an invaluable resource for identifying cell types that are particularly responsive to fever-range heat stress, as well as biomarkers and therapeutic targets for the diagnosis and treatment of disorders associated with fever (Fig. 2T).

A physiological or pathological fever directly affects health and induces fever-associated disorders in tissues throughout the body. To determine cell types that are particularly susceptible to heat stress, we obtained isogenic stem cells and terminally differentiated derivatives using directed differentiation to provide a valuable platform for evaluating and comparing the effects of 39 °C hyperthermia. Through the comparative analysis of heat shock-related DEGs across seven isogenic cell types, we found that most hyperthermia-associated DEGs were cell type-specific. We observed relatively mild responses at the transcriptional level in cell types of the nervous system, as observed in hNSCs and hNeurons. In fact, brain damage from a fever is generally evident when the temperature is over 40 °C (Kim et al., 1996). However, the developing brain is especially sensitive to hyperthermia (White et al., 2007), as children whose mothers have any type of fever during pregnancy may have slightly increased odds of developing an autism spectrum disorder (Hornig et al., 2018). The inconsistency may attribute to the duration of hyperthermia and the developmental stage of human neurons. Further investigations besides RNA-seq may deepen our understanding of fever-induced neurological damage. By contrast, we found that cells comprising the vascular structure, including hVECs (in the intima of blood vessels) and hMSCs (in the adventitia of blood vessels), were more sensitive to fever, implying heat exposure leads to an acute response in the vasculature. It has been reported that heat stress increases the levels of HSP family protein and circulating nitric oxide (NO) through increased eNOS activity, which may elicit arterial adaptation and promote vascular protection (Cheng and MacDonald, 2019). The differential response of cell lineages to fever may reflect a dedicated, compensatory mechanism to mitigate heat-induced damage, otherwise eliminate the unrepaired cells at the physiological level. Furthermore, we have discovered a novel role for CDC20 as a potential mediator of fever-range hyperthermia-induced cell cycle arrest in hMSCs. Therefore, the generation of CDC20-engineered functionally enhanced stem cells counteracting heat-induced damages may serve as a new potential stem cell-based therapeutic strategy against hyperthermia-related disorders, such as infection and inflammatory diseases. In addition to physiological and pathological fever, the human body may also be exposed to extreme heat environments. Therefore, this study not only provides information that may help to develop cell/tissue-specific interventions that therapeutically target heat shock-related diseases, but also supports the development of protective strategies for high-heat environments.

Due to the anatomical and physiological similarity between pigs and humans, it has been reported that human-pig chimeras may produce human organs for transplantation (Wu et al., 2017). However, the efficiency of chimerism is not as high as for mouse-rat interspecies chimeras (Wu et al., 2017). This limits the application in the field of regenerative medicine. In addition to the evolutionary distance between human and pig, differences in body temperature between pigs (39 °C) and humans may underlie the lack of success. Our results imply that heat stress may induce increased apoptosis in hESCs in chimera systems, resulting in compromised self-renewal and differentiation. In addition, we found that 39 °C hyperthermia is deleterious to adult human stem cells and their differentiated derivatives, which may dampen both the growth and the functional maturation of a human organ generated in a chimeric system. Our data may indicate heat-sensitive or heat-resistant factors that can be targeted by genetic or pharmacological interventions to promote organ growth for transplantation.

Our study provides a transcriptional profiling atlas of various human stem cells and their derivatives under febrile temperature stress. The data reveals molecular modulation related to fever-range heat stress and subsequent cytotoxicity. However, there are a number of concerns to be addressed. To begin with, it is worth noting that naïve (pre-implantation) but not primed (post-implantation) pluripotent stem cells exist under *in vivo* physiological conditions. The responses to heat stress in naïve hESCs need further investigations. Moreover, it is necessary to expand examined cell and tissue types in order to better understand the effects of heat stress in a broader range of cell contexts. In conclusion, our findings advance understanding of how different tissues and organs respond to a high-temperature environment, paving a path toward future development of new treatments against fever-related disorders.

## Supplementary Information

Below is the link to the electronic supplementary material.

Supplementary file1 (PDF 2434 kb)

Supplementary file2 (XLSX 383 kb)

Supplementary file3 (XLSX 477 kb)
