# Supplementary material for: Hyperthermia differentially affects specific human stem cells and their differentiated derivatives
Source: Protein Cell. 2021 Nov 1;13(8):615–22. doi: 10.1007/s13238-021-00887-y (PMC9232687; doi:10.1007/s13238-021-00887-y)
Supplement: Supplementary file 1 — Supplementary file1 (PDF 2434 kb) [file 13238_2021_887_MOESM1_ESM.pdf]

Supplementary Materials

Materials and Methods

Cell culture

hESCs (Line H9, WA09, WiCell Research Institute, Inc.) were cultured on feeder cells of Mitomycin C (MMC) (Selleck)-inactivated mouse embryonic fibroblast (MEF) in DMEM/F12 medium (Gibco) containing 20% KnockOut Serum Replacement (Gibco), 0.1 mM non-essential amino acids (NEAA, Gibco), 2 mM GlutaMAX (Gibco), 1% penicillin/streptomycin (Gibco), 55 μM β-mercaptoethanol (Thermo Fisher Scientific), and 10 ng/mL FGF2 (Joint Protein Central, JPC) or on Matrigel (Corning) in mTeSR medium (STEMCELL Technologies). hMSCs were cultured in α-MEM medium (Gibco) supplemented with 10% fetal bovine serum (FBS, Gibco), 0.1 mM NEAA (Gibco), 1% penicillin/streptomycin (Gibco), and 1 ng/mL FGF2 (JPC). hVSMCs were cultured in N2B27 medium supplemented with 50% Neurobasal (Gibco), 50% DMEM/F12 medium (Gibco), 1× N2 (Gibco), 1× B27 (Gibco), 10 ng/mL PDGF-AB (Peprotech), 55 μM β-mercaptoethanol (Gibco) and 1% penicillin/streptomycin (Gibco). hVECs were maintained in EGM-2 (Lonza) medium supplemented with 50 ng/mL VEGF165 (HumanZyme), 20 ng/mL FGF2 (JPC) and 10 nM SB431542 (Selleck). hCardiomyocytes (hCMs) were cultured in RPMI1640 medium (Gibco) supplemented with S12 (plus insulin). The S12 medium was an albumin-free and chemically defined supplement for cardiac differentiation including fatty acid, protein, chemicals, and antioxidants (Jiang et al., 2012). hNSCs were grown in neural stem cell maintenance medium (NSMM) containing 50% Advanced DMEM/F12 (Gibco), 50% Neurobasal (Gibco), 2 mM GlutaMAX, 0.1 mM NEAA (Gibco), 1% penicillin/streptomycin (Gibco), 1× N2 (Gibco), 1× B27 (Gibco), 10 ng/mL human leukemia inhibitory factor (hLIF, Millipore), 2 μM SB431542 (Selleck) and 3 μM CHIR99021 (Selleck). hNeurons were maintained in Advanced DMEM/F12 medium supplemented with 1× N2, 1× B27, 200 μM Ascorbic acid (Sigma), 400 μM dbcAMP (Sigma), 10 ng/mL GDNF (Peprotech) and 10 ng/mL of BDNF (Peprotech), 1% penicillin/streptomycin (Gibco).

Generation of hVECs from hESCs

hVECs were differentiated from H9-hESCs as previously described (Wang et al., 2018). Briefly, the H9-ESCs were firstly picked onto Matrigel-coated plates in mTeSR medium. The next day, mTeSR medium was switched to EGM-2 medium (Lonza) supplemented with 25 ng/mL BMP4 (R&D), 3 μM CHIR99021 (Selleck), 3 μM IWP2 (Selleck) and 4 ng/mL FGF2 (JPC). After three days, the culture medium was replaced with EGM-2 medium supplemented with 50 ng/mL VEGF165 (HumanZyme), 20 ng/mL FGF2 (JPC), and 10 ng/mL IL-6 (Peprotech) for another three days. The cultures were then split with Accumax (Millipore). To purify hVECs, cells were stained with anti-human CD144-APC (BD, 561567) and anti-human CD201-PE (BD, 351904) antibodies and then sorted using a flow cytometer (BD, Aria II) (Ling et al., 2019; Wang et al., 2018; Yan et al., 2019). For all febrile temperature experiments, hVECs at passage 2 or 3 were firstly plated in two sets at 37 ºC for 24 h. Afterward, one set of cells was

transferred to 39 ºC and cultured for additional 48 h, while the other set was maintained at 37 ºC.

Generation of hVSMCs from hESCs

hVSMC induction was performed according to a previous study (Ling et al., 2019). Briefly, H9-hESCs were split into single cells with TrypLE (Thermo Fisher Scientific) and about 3×10^5 cells for each well were cultured onto Matrigel-coated 6-well plates in mTeSR medium supplemented with 10 μM Y-27632 (Selleck) at the first day. Cultures were then maintained in N2B27 medium supplemented with 25 ng/mL BMP4 (R&D), 8 μM CHIR99021 (Selleck). After three days, cells were then cultured in N2B27 medium with 10 ng/mL PDGF-AB (Peprotech) and 2 ng/mL Activin A (HumanZyme) for another two days and the medium was changed every day. The cells were then stained with anti-human CD140b-PE (BD biosciences, 558821) antibody and sorted by flow cytometry (BD, Aria II) (Ling et al., 2019; Wang et al., 2018). For all febrile temperature experiments, hVSMCs at passage 2 or 3 were first plated in two sets at 37 ºC for 24 h. Afterward, one set of cells was transferred to 39 ºC and cultured for another 48 h, and the other set was maintained at 37 ºC.

Generation of hMSCs from hESCs

Differentiation of hESCs into hMSCs was performed following a published protocol (Lei et al., 2021). In brief, embryoid bodies (EBs) were plated on Matrigel-coated plates in hMSC differentiation medium consisting of 90% α-MEM, 10% FBS (Gibco), 0.1 mM NEAA (Gibco), 1% penicillin/streptomycin (Gibco), 10 ng/mL FGF2 (JPC) and 5 ng/mL TGFβ (HumanZyme). Approximately 10 days later, fibroblast-like cells appeared. These cells were further maintained in hMSC culture medium. hMSCs were sorted as CD73, CD90 and CD105 tri-positive cells using fluorescence-activated cell sorting (FACS) (BD, Aria II). Antibodies used were as follow: anti-human CD73-PE (BD Biosciences, 550257), anti-human CD90-FITC (BD Biosciences, 555595), anti-human CD105-APC (BD Biosciences, 323208), anti-human CD34-PE (BD Biosciences, 555822), anti-human CD43-FITC (BD Biosciences, 580198), and anti-human CD45-FITC (BD Biosciences, 555482). Anti-IgG-PE (BD Biosciences, 555749), anti-IgG-FITC (BD Biosciences, 555748), and anti-IgG-APC (BD Biosciences, 555751) antibodies were used as isotype controls. For febrile temperature experiments, hMSCs at passage 4 or 5 were first plated in two sets at 37 ºC for 24 h. Afterward, one set of cells was transferred to 39 ºC and cultured for another 48 h, and the other set was maintained at 37 ºC.

Generation of hCMs from hESCs

Differentiation of hESCs into hCMs was performed as previously described (Jiang et al., 2012; Lee et al., 2017). hESCs were split into small clusters with 0.5 mM EDTA (Sigma, EDS-500G) and plated onto Matrigel-coated 24-well plates in mTESR medium. Differentiation of hESCs into hCMs was carried out when the density of hESCs reached a confluency of 80% to 90%. Culture medium was switched to differentiation medium constituting with RPMI1640 and S12 (without insulin). 3 μM CHIR99021 was added

at day 0. After 24 h, the medium was changed back to differentiation medium. 5 μM IWR-1 was added at days 3-5. From day 5 to day 11, the differentiation medium was changed to RPMI1640 supplemented with S12 (plus insulin). To purify the hCMs, the medium was changed to RPMI1640 (without glucose) supplemented with S12 (plus insulin) and lactate every two days for three times starting from day 12. For all febrile temperature experiments, one set of hCMs (about at 21 days) was transferred to 39 ºC and cultured for additional 48 h, and another set was maintained at 37 ºC.

Generation of hNSCs from hESCs

Differentiation of hESCs into hNSCs was described previously (Zhang et al., 2019). H9-hESCs were picked onto MEF feeder cells in hESC culture medium for about 2 days. The culture medium was then switched to Neural Induction Medium 1 containing 50% Neurobasal (Gibco), 50% Advanced DMEM/F12(Gibco), 2 mM GlutaMAX (Gibco), 0.1 mM NEAA (Gibco), 1% penicillin/streptomycin (Gibco), 1×B27 (Gibco), 1×N2 (Gibco), 10 ng/mL hLIF (Millipore), 3 μM SB431542 (Selleck), 4 μM CHIR99021 (Selleck), 2 μM dorsomorphin (Sigma) and 0.1 μM Compound E (EMD Chemicals, Inc.). After 2 days, culture medium was replaced with Neural Induction Medium 2 containing 50% Neurobasal, 50% Advanced DMEM/F12, 2 mM GlutaMAX, 0.1 mM NEAA (Gibco), 1% penicillin/streptomycin (Gibco), 1×B27, 1×N2, 10 ng/mL hLIF, 3 μM SB431542 and 4 μM CHIR99021 and 0.1 μM Compound E for another five days. The cells were then passaged onto Matrigel-coated plates with Accumax (Millipore) and cultured in NSMM. For febrile temperature experiments, hNSCs at passage 6 or 7 were first plated in two sets at 37 ºC for 24 h. Afterward, one set of cells was transferred to 39 ºC and cultured for another 48 h and the other set was maintained at 37 ºC.

Neuronal differentiation from hNSCs

Neuronal differentiation from hNSCs was as previously described (Zhang et al., 2019). Firstly, hNSCs were plated onto Matrigel-coated 24-well plates at a density of 3×10^4 cells per well, and maintained in NSMM for 1-3 days. The cultures were then maintained in differentiation medium with Advanced DMEM/F12, 1× N2, 1× B27, 200 μM Ascorbic acid (Sigma), 400 μM dbcAMP (Sigma), 10 ng/mL GDNF (Peprotech) and 10 ng/mL of BDNF (Peprotech), 1% penicillin/streptomycin for two days before 20 μg/mL laminin (Sigma) was added to further facilitate differentiation. About 14 days later, differentiated hNeurons were immunostained with an antibody against the neuron marker MAP2. For febrile temperature experiments, one set of hNeurons (about at 21 days) was transferred to 39 ºC and cultured for another 48 h and the other set was maintained at 37 ºC.

Apoptosis analysis

Cell apoptosis assays were performed using Annexin V-EGFP Apoptosis Detection Kit (Vigrous Biotechnology) according to the manufacturer's instructions. Cells were cultured at 37 ºC or 39 ℃ in 5% CO_2 for 48 h, respectively. Then the cells were stained with Annexin V-EGFP Apoptosis Detection Kit and analyzed by flow cytometry (BD

LSRForteas). The percentage of apoptotic cells were analyzed by FlowJo_V10 software.

Immunofluorescence microscopy

Cells were fixed with 4% paraformaldehyde at room temperature for 30 min, washed with PBS, permeabilized with 0.4% Triton X-100 in PBS at room temperature for another 30 min and then blocked with 10% donkey serum (Jackson ImmunoResearch Labs) in PBS at room temperature for 1 h. The cells were then incubated with primary antibodies in blocking solution at 4 °C overnight followed by incubation with corresponding fluorescent secondary antibodies and Hoechst 33342 (Thermo Fisher Scientific) at room temperature for 1 h. Images were captured using a confocal microscope (Leica SP5). The primary antibodies used were as follows (company, catalogue number): anti-OCT4 (Santa Cruz, sc-5279), anti-SOX2 (Santa Cruz, sc-17320), anti-NANOG (Abcam, ab21624), anti-Ki67 (ZSGB-Bio, ZM-0166), anti-PAX6 (BioLegend, 901301), anti-Nestin (BD, 560422), anti-MAP2 (Sigma, M4403), anti-CD144 (Cell Signaling Technology, 2158S), anti-CD31 (BD, 555445), anti-eNOS (BD, 61029), anti-SM22 (Abcam, ab14106), anti-Calponin (DAKO, M3556), anti-Cardiac Troponin T (cTnT) (ab45932), anti-γH2AX (Millipore, 05-636), anti-53BP1 (Bethyl Laboratories, A300-273A).

Cell cycle analysis

Cells were collected and fixed in 70% ice-cold ethanol at -20 °C overnight. Next day, the cells were stained with 0.2 mg/mL RNase and 0.02 mg/ml propidium iodide (PI) at 37 °C for 30 min. Samples were then analyzed using BD LSRFortesa, and cell-cycle phase distributions were analyzed by ModFit software.

Lentivirus production

To package the lentiviruses, HEK293T cells were co-transfected with corresponding lentiviral vectors, psPAX2 (Addgene, #12260) and pMD2G (Addgene, #12259) using Lipofectamine^{TM} 3000 Transfection Reagent (Thermo Fisher Scientific). Viral particles were collected by ultracentrifugation at 19,400 × *g* at 4 °C for 2.5 h.

Lentiviral CRISPR/Cas9-mediated gene knockdown and activation

Lentiviral CRISPR/Cas9-mediated gene knockdown and activation were performed as previously described (Wu et al., 2020). To construct CRISPR/Cas9-mediated gene knockdown lentiviral vectors, the sgRNA targeting CDC20 (corresponding sgRNA oligonucleotide: TTCCCTGCCAGACCGTATCC) was inserted into the cloning site of lentiCRISPRv2 (Addgene, #52961). LentiCRISPRv2-NTC (non-targeting control) was used as negative control. To induce endogenous CDC20 expression, sgRNA (corresponding sgRNA oligonucleotide: CAGTACTAGTCCTCTGGCGC) targeting the transcriptional start site (TSS) of CDC20 was inserted into lentiSAM v2 vector (Addgene, #75112). LentiSAM v2-NTC (non-targeting control) was used as negative control. For lentiviral CRISPR/Cas9-mediated gene knockdown, Lenti-CRISPRv2-CDC20 were transduced into hMSCs. 48 h post-transfection, the cells were selected with puromycin (Thermo Fisher Scientific). For the induction of endogenous

expression of CDC20, hMSCs were co-transduced with the LentiSAMv2 and LentiMPH v2 to transcriptionally activate the expression of CDC20. 48 h later, the cells were selected with blasticidin and hygromycin for 7 days.

Western blotting

Cells were lysed in lysis buffer (Millipore) with protease inhibitor cocktail (Roche) for 30 min. Then cell lysates were centrifuged at 17,000 × *g* for 30 min. A following BCA kit was used for protein quantification. 20 µg of cell lysate was loaded onto SDS-PAGE gels for electrophoresis and electrotransferred to PVDF membranes (Millipore). After blocking with 5% (w/v) nonfat powdered milk (BBI Life Sciences), the membrane was incubated with primary antibodies overnight at 4 °C and HRP-conjugated secondary antibodies followed at room temperature for 1 h. Finally, imaging was performed with the ChemiDoc XRS system (Bio-Rad). The primary antibodies used for western blotting in this study were anti-HSP90AA1 (Abcam, ab79849), anti-GAPDH (Sigma, G8795), and anti-CDC20 (Santa Cruz, 13162).

RNA-seq library construction and sequencing

Briefly, total RNAs were extracted using TRIzol Reagent, and genomic DNA was removed using a DNA-free Kit. Library preparation was conducted using a NEBNext® UltraTM Directional RNA Library Prep Kit for Illumina (New England Biolabs). Quality control and sequencing on Illumina HiSeq X Ten platforms were performed by Novogene Bioinformatics Technology Co., Ltd.

RNA-seq data processing

RNA-seq data processing was performed as previously described (Bi et al., 2020). We first inspected RNA-seq read quality using FASTQC (v0.11.6), low-quality reads and adaptors were then trimmed using TrimGalore (v0.4.4_dev). The remaining clean reads were mapped to the UCSC human hg19 genome using HISAT2 software (v2.1.0). Reads on each annotated gene were counted using HTSeq (v0.6.1). Differentially expressed genes (DEGs) were calculated using DESeq2 with a cutoff adjust *P*-value less than 0.05 and $|Log_2$ (fold change)$|$ more than 0.5. Gene Ontology (GO) term enrichment analysis was performed using Metascape with *P*-value less than 0.05 (http://metascape.org/gp/index.html). The principal component analysis (PCA) and Euclidian distance were performed using R based on Log_2 (FPKM + 1). Gene lists of "cell cycle", "DNA repair" and "positive regulation of cell death" were obtained from Gene Ontology Resource. Core regulatory transcription factors were predicted based on the DEGs under febrile temperature (39 °C) in seven cell types. Transcription factor-binding motifs were identified by using R/Bioconductor package RcisTarget (v1.10.0). The DEGs and GO terms are listed in Supplementary Table S1 and S2.

Statistical analysis

Data are presented as the mean ± SEM. GraphPad Prism software was used to perform a two-tailed Student's t-test. Statistical significance is presented as * $P < 0.05$, ** $P < 0.01$ and *** $P < 0.001$.

Data availability

The sequencing data have been deposited in the Genome Sequence Archive (GSA) in the National Genomics Data Center, Beijing Institute of Genomics (China National Center for Bioinformation) of the Chinese Academy of Sciences, under accession number HRA000922 that are publicly accessible at http://bigd.big.ac.cn/gsa-human. The data can also be accessed via an interactive user-friendly webtool at Aging Atlas (https://bigd.big.ac.cn/aging/index) (Aging Atlas, 2021).

References

Aging Atlas, C. (2021). Aging Atlas: a multi-omics database for aging biology. Nucleic acids research *49*, D825-D830.

Bi, S., Liu, Z., Wu, Z., Wang, Z., Liu, X., Wang, S., Ren, J., Yao, Y., Zhang, W., Song, M.*, et al.* (2020). SIRT7 antagonizes human stem cell aging as a heterochromatin stabilizer. Protein Cell *11*, 483-504.

Jiang, J., Han, P., Zhang, Q., Zhao, J., and Ma, Y. (2012). Cardiac differentiation of human pluripotent stem cells. J Cell Mol Med *16*, 1663-1668.

Lee, W.H., Chen, W.Y., Shao, N.Y., Xiao, D., Qin, X., Baker, N., Bae, H.R., Wei, T.T., Wang, Y., Shukla, P.*, et al.* (2017). Comparison of Non-Coding RNAs in Exosomes and Functional Efficacy of Human Embryonic Stem Cell- versus Induced Pluripotent Stem Cell-Derived Cardiomyocytes. Stem Cells *35*, 2138-2149.

Lei, J., Jiang, X., Li, W., Ren, J., Wang, D., Ji, Z., Wu, Z., Cheng, F., Cai, Y., Yu, Z.R.*, et al.* (2021). Exosomes from antler stem cells alleviate mesenchymal stem cell senescence and osteoarthritis. Protein & cell.

Ling, C., Liu, Z., Song, M., Zhang, W., Wang, S., Liu, X., Ma, S., Sun, S., Fu, L., Chu, Q.*, et al.* (2019). Modeling CADASIL vascular pathologies with patient-derived induced pluripotent stem cells. Protein Cell *10*, 249-271.

Wang, P., Liu, Z., Zhang, X., Li, J., Sun, L., Ju, Z., Li, J., Chan, P., Liu, G.H., Zhang, W.*, et al.* (2018). CRISPR/Cas9-mediated gene knockout reveals a guardian role of NF-kappaB/RelA in maintaining the homeostasis of human vascular cells. Protein & cell *9*, 945-965.

Wu, Z., Shi, Y., Lu, M., Song, M., Yu, Z., Wang, J., Wang, S., Ren, J., Yang, Y.G., Liu, G.H.*, et al.* (2020). METTL3 counteracts premature aging via m6A-dependent stabilization of MIS12 mRNA. Nucleic acids research *48*, 11083-11096.

Yan, P., Li, Q., Wang, L., Lu, P., Suzuki, K., Liu, Z., Lei, J., Li, W., He, X., Wang, S.*, et al.* (2019). FOXO3-Engineered Human ESC-Derived Vascular Cells Promote Vascular Protection and Regeneration. Cell stem cell *24*, 447-461 e448.

Zhang, X., Liu, Z., Liu, X., Wang, S., Zhang, Y., He, X., Sun, S., Ma, S., Shyh-Chang, N., Liu, F.*, et al.* (2019). Telomere-dependent and telomere-independent roles of RAP1 in regulating human stem cell homeostasis. Protein Cell *10*, 649-667.

Supplementary Figure Legends

Figure S1. Generation and characterization of human stem cells and their derivatives

(A) hESC pluripotency markers shown by immunofluorescence staining. Scale bar, 25 μm.

(B) hVEC-specific markers CD31, CD144 and eNOS shown by immunofluorescence staining. Scale bar, 25 μm.

(C) VSMC-specific markers, SM22 and Calponin shown by immunofluorescence staining. Scale bar, 25 μm.

(D) Flow cytometric analysis of hMSC-specific markers, CD90, CD73 and CD105 (left) and hMSC-irrelevant markers, CD34, CD43 and CD45 (right).

(E) hCM-specific marker cTnT shown by immunofluorescence. Scale bar, 10 μm.

(F) hNSC-specific markers, SOX2, Nestin and PAX6 shown by immunofluorescence. Scale bar, 25 μm.

(G) Phase-contrast images of hNeurons to the left. Scale bar, 200 μm. hNeuron-specific marker MAP2 shown by immunofluorescence to the right. Scale bar, 50 μm.

(H) Heatmap showing the relative transcriptional expression for cell type-specific genes. Representative Gene Ontology (GO) terms and corresponding P values for each set of cell type-specific genes are shown to the right.

(I) Heatmap showing the Euclidian distance between replicates of indicated groups across seven cell types.

(J) Principal component analysis showing high reproducibility of transcriptome expression profiles between replicates.

(K) Volcano plots showing the differentially expressed genes between the cells under febrile temperature (39 °C) culture condition and their counterparts under control (37 °C) culture condition in seven cell types.

Figure S2. The transcriptomic profiles of human stem cells and their derivatives under 39°C heat stress

(A) UpSet plots showing the numbers of upregulated (top) and downregulated (bottom) hyperthermia DEGs shared across seven cell types.

(B) Dot plot showing the hyperthermia DEGs related to positive regulation of cell death across seven cell types.

(C-D) Dot plots showing downregulated hyperthermia DEGs related to cell cycle (C), DNA repair (D) across seven cell types.

Figure S3. The transcriptomic profile alterations upon genetical manipulation of CDC20 in hMSCs

(A) UpSet plots showing the numbers of cell type-specific hyperthermia DEGs in seven cell types. Upregulated genes (top); downregulated genes (bottom).

(B-C) Dot plots showing the potential upstream transcriptional regulatory factors for cell type-specific upregulated (B) and downregulated (C) hyperthermia-associated DEGs.

(D) Ridge plots showing the shift in expression levels of cell type-specific hyperthermia

DEGs related to cell cycle in seven cell types.

(E) Bar plot showing the percentage of cell cycle-related genes to total downregulated hyperthermia-associated DEGs in each cell type.

(F) Schematic of CRISPR/Cas9 mediated gene knockout or CRISPR-dCas9 mediated transcriptional activation systems. NTC, non-targeting control.

(G) Representative immunofluorescence of Ki67 in hMSCs transduced with sgNTC or CDC20-targeting knockout sgRNA under control (37 °C) and febrile temperature (39 °C) culture conditions. Scale bar, 20 μm.

(H) Flow cytometric analysis of apoptosis in hMSCs transduced with sgNTC or CDC20-targeting knockout sgRNA under control (37 °C and febrile temperature (39 °C) culture conditions.

(I) Representative immunofluorescence of Ki67 in hMSCs transduced with sgNTC or CDC20-targeting activation sgRNA under control (37 °C) and febrile temperature (39 °C) culture conditions. Scale bar, 20 μm.

(J) Flow cytometric analysis of apoptosis in hMSCs transduced with sgNTC or CDC20-targeting activation sgRNA under control (37 ℃) and febrile temperature (39 ℃) culture conditions.

(K) Heatmap showing the Euclidian distance between replicates among the indicated groups.

(L) Heatmaps showing the expression level of hyperthermia-associated DEGs in sgNTC transduced hMSCs (left) and DEGs upon CDC20 knockdown in hMSCs at 37 ℃ (right).

(M) Heatmap showing the Euclidian distance between replicates among the indicated groups.

(N) Heatmaps showing the expression level of hyperthermia-associated DEGs in sgNTC transduced hMSCs (left) and DEGs upon CDC20 activation in hMSCs at 39 ℃ (right).

Supplementary Table Legends

Table S1. Hyperthermia-associated differentially expressed genes (DEGs) and the corresponding Gene Ontology (GO) terms across seven cell types.

Table S2. DEGs upon knockdown or activation of CDC20 in hMSCs and the shared GO terms between different conditions.

Figure S1

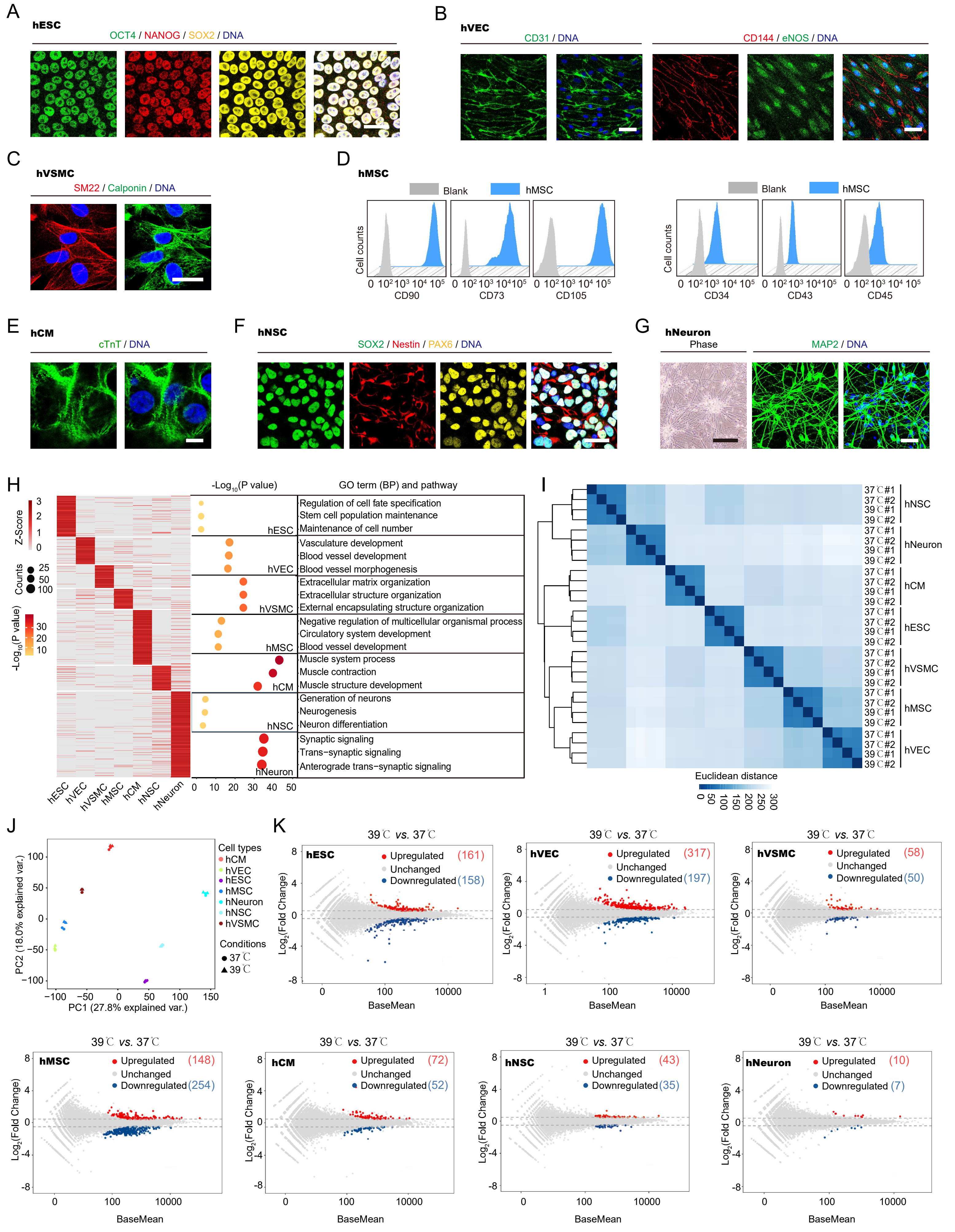

A
hESC
OCT4 / NANOG / SOX2 / DNA
B
hVEC
CD31 / DNA
CD144 / eNOS / DNA
C
hVSMC
SM22 / Calponin / DNA
D
hMSC
Blank
hMSC
Cell counts
CD90
CD73
CD105
CD34
CD43
CD45
E
hCM
cTnT / DNA
F
hNSC
SOX2 / Nestin / PAX6 / DNA
G
hNeuron
Phase
MAP2 / DNA
H
Z-Score
Counts
-Log10(P value)
GO term (BP) and pathway
Regulation of cell fate specification
Stem cell population maintenance
Maintenance of cell number
Vasculature development
Blood vessel development
Blood vessel morphogenesis
Extracellular matrix organization
Extracellular structure organization
External encapsulating structure organization
Negative regulation of multicellular organismal process
Circulatory system development
Blood vessel development
Muscle system process
Muscle contraction
Muscle structure development
Generation of neurons
Neurogenesis
Neuron differentiation
Synaptic signaling
Trans−synaptic signaling
Anterograde trans−synaptic signaling
hESC
hVEC
hVSMC
hMSC
hCM
hNSC
hNeuron
I
Euclidean distance
hNSC
hNeuron
hCM
hESC
hVSMC
hMSC
hVEC
37℃#1
37℃#2
39℃#1
39℃#2
J
PC1 (27.8% explained var.)
PC2 (18.0% explained var.)
Cell types
hCM
hVEC
hESC
hMSC
hNeuron
hNSC
hVSMC
Conditions
37℃
39℃
K
39℃ vs. 37℃
hESC
Upregulated (161)
Unchanged
Downregulated (158)
hVEC
Upregulated (317)
Unchanged
Downregulated (197)
hVSMC
Upregulated (58)
Unchanged
Downregulated (50)
hMSC
Upregulated (148)
Unchanged
Downregulated (254)
hCM
Upregulated (72)
Unchanged
Downregulated (52)
hNSC
Upregulated (43)
Unchanged
Downregulated (35)
hNeuron
Upregulated (10)
Unchanged
Downregulated (7)
Log2(Fold Change)
BaseMean

Figure S2

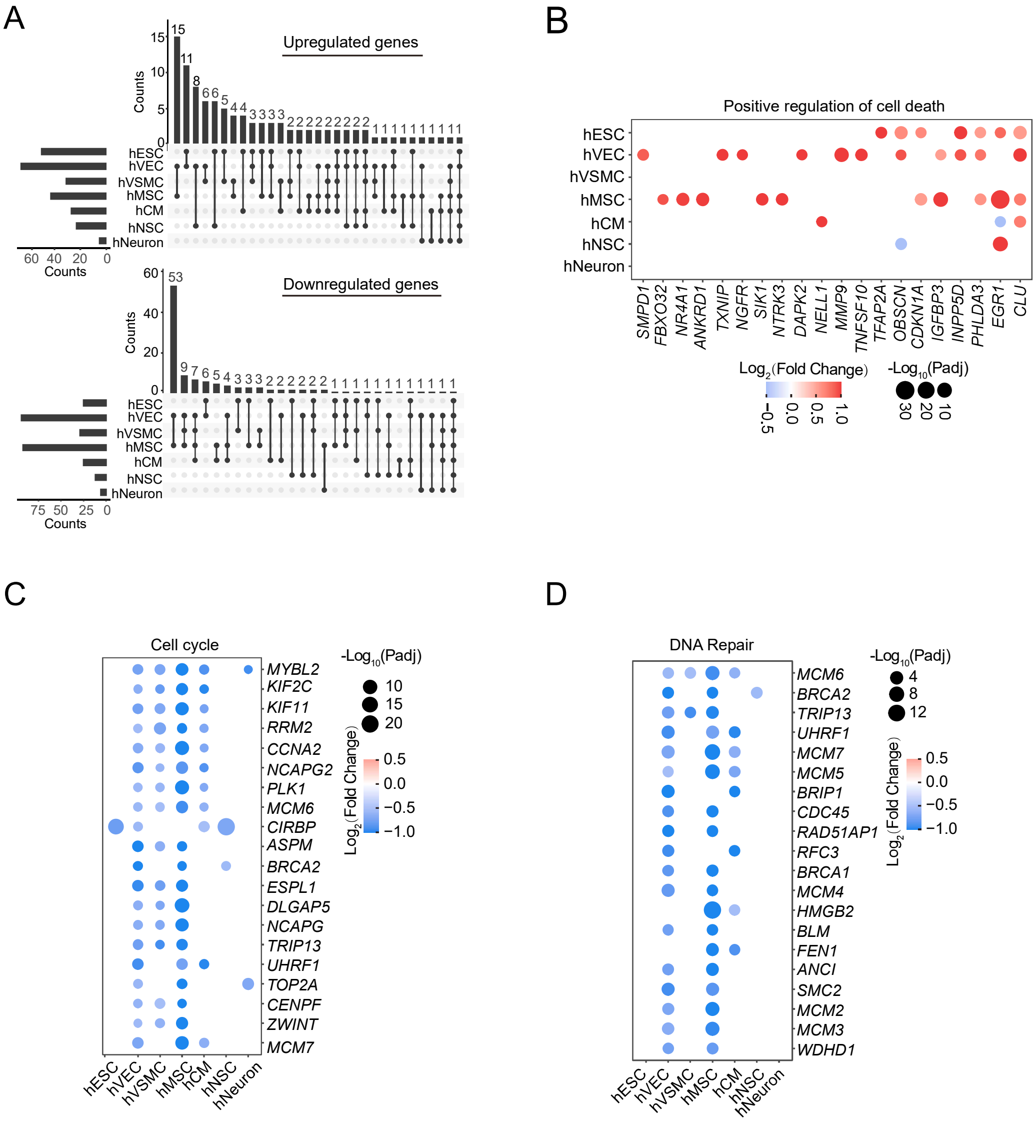
A
Upregulated genes
Downregulated genes
Counts
hESC
hVEC
hVSMC
hMSC
hCM
hNSC
hNeuron
B
Positive regulation of cell death
SMPD1
FBXO32
NR4A1
ANKRD1
TXNIP
NGFR
SIK1
NTRK3
DAPK2
NELL1
MMP9
TNFSF10
TFAP2A
OBSCN
CDKN1A
IGFBP3
INPP5D
PHLDA3
EGR1
CLU
Log2(Fold Change)
-Log10(Padj)
C
Cell cycle
MYBL2
KIF2C
KIF11
RRM2
CCNA2
NCAPG2
PLK1
MCM6
CIRBP
ASPM
BRCA2
ESPL1
DLGAP5
NCAPG
TRIP13
UHRF1
TOP2A
CENPF
ZWINT
MCM7
D
DNA Repair
MCM6
BRCA2
TRIP13
UHRF1
MCM7
MCM5
BRIP1
CDC45
RAD51AP1
RFC3
BRCA1
MCM4
HMGB2
BLM
FEN1
ANCI
SMC2
MCM2
MCM3
WDHD1

Figure S3

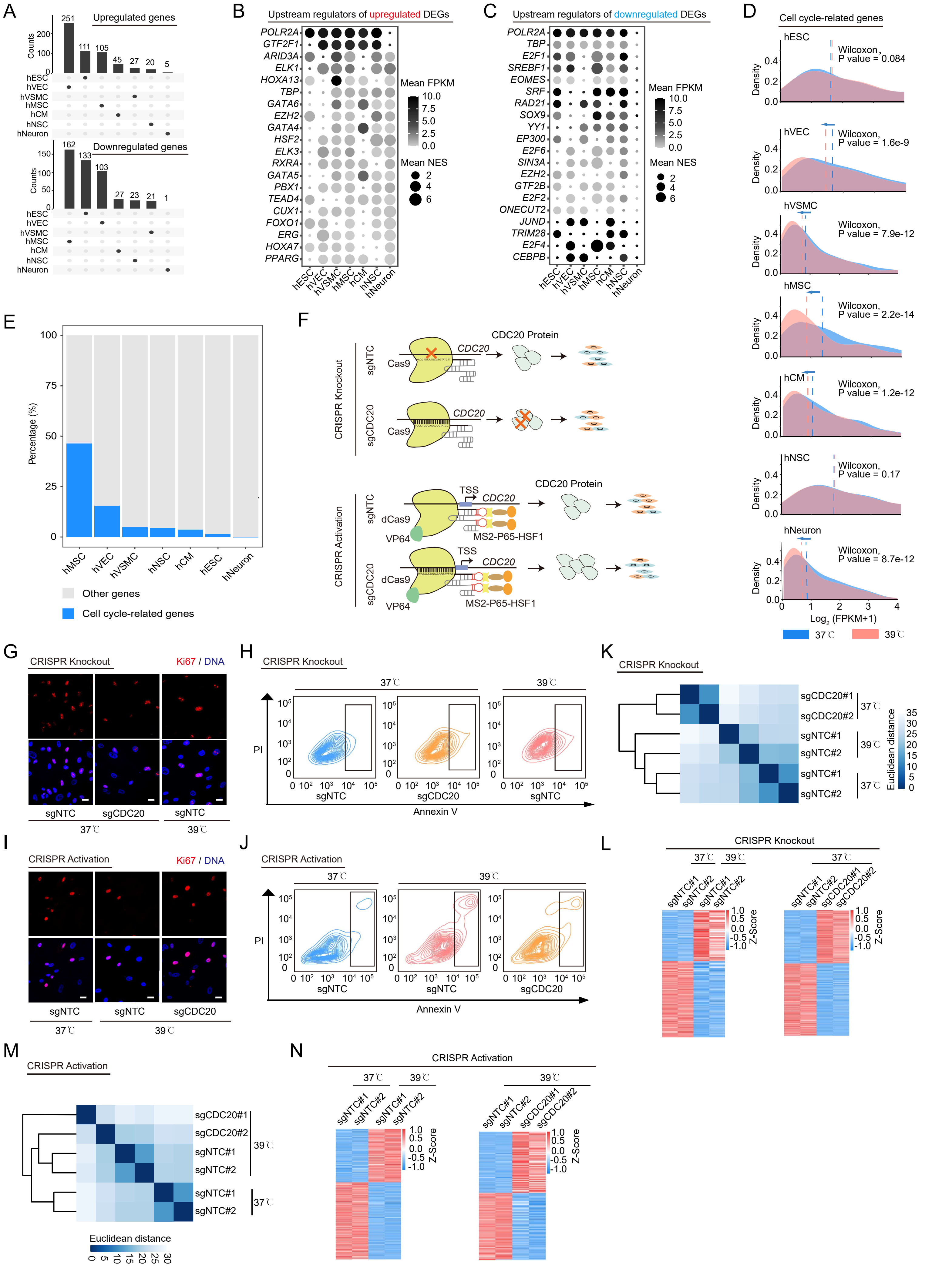

A
Upregulated genes
Downregulated genes
Counts
hESC
hVEC
hVSMC
hMSC
hCM
hNSC
hNeuron
B
Upstream regulators of upregulated DEGs
Mean FPKM
Mean NES
C
Upstream regulators of downregulated DEGs
D
Cell cycle-related genes
Density
Log2 (FPKM+1)
37℃
39℃
E
Percentage (%)
Other genes
Cell cycle-related genes
F
CRISPR Knockout
CRISPR Activation
CDC20 Protein
G
CRISPR Knockout
Ki67 / DNA
H
CRISPR Knockout
Annexin V
I
CRISPR Activation
J
CRISPR Activation
K
CRISPR Knockout
Euclidean distance
L
CRISPR Knockout
Z-Score
M
CRISPR Activation
N
CRISPR Activation
